# Applications of Heterogeneous Photocatalysis to the Degradation of Oxytetracycline in Water: A Review

**DOI:** 10.3390/molecules27092743

**Published:** 2022-04-24

**Authors:** Renato Pelosato, Isabella Bolognino, Francesca Fontana, Isabella Natali Sora

**Affiliations:** INSTM and Department of Engineering and Applied Sciences, University of Bergamo, 24044 Dalmine, Italy; renato.pelosato@unibg.it (R.P.); isabella.bolognino@unibg.it (I.B.); francesca.fontana@unibg.it (F.F.)

**Keywords:** oxytetracycline, photocatalytic wastewater treatment, advanced oxidation processes

## Abstract

Photocatalytic processes are being studied extensively as potential advanced wastewater treatments for the removal of pharmaceuticals, pesticides and other recalcitrant micropollutants from the effluents of conventional wastewater treatment plants (WWTPs). Oxytetracycline (OTC) is a widespread antibiotic which is frequently detected in surface water bodies as a recalcitrant and persistent micropollutant. This review provides an update on advances in heterogeneous photocatalysis for the degradation of OTC in water under UV light, sunlight and visible-light irradiation. Photocatalysts based on pure semiconducting oxides are rarely used, due to the problem of rapid recombination of electron–hole pairs. To overcome this issue, a good strategy could be the coupling of two different semiconducting compounds with different conduction and valence bands. Several methods are described to enhance the performances of catalysts, such as doping of the oxide with metal and/or non-metal elements, surface functionalization, composites and nano-heterojunction. Furthermore, a discussion on non-oxidic photocatalysts is briefly provided, focusing on the application of graphene-based nanocomposites for the effective treatment of OTC.

## 1. Introduction

The problem of surface water pollution is of increasing concern worldwide, due to the huge amount of chemicals (drugs, pesticides, cosmetics, food additives, plastics additives, etc.) present in wastewater as a consequence of human activities. Contaminants may come from a wide variety of point or non-point sources and be more or less dangerous depending on their physiological effects on humans and animals, their abundance and their persistence in the environment. In recent years, the problem of micropollutants, often constituted by substances which are only partially removed by treatment plants and thus persist in traces in water bodies, has raised increasing global concern and scientific interest and has been challenged by a variety of advanced decontamination techniques [1]. Among persistent micropollutants, an important role is attributed to residues of drugs (and their metabolites), coming from human consumption as well as from agricultural activities and livestock farms. Some of the most utilized drugs, such as antibiotics, tend to persist in water and be the source of potential damage to humans and the environment. The occurrence of antibiotics in the environment may have an adverse impact on the ecosystem and human health through the development of antibiotic-resistant bacteria and pathogens [2]. Antibiotics may also negatively affect soil microbial activity, enzyme activity, plant growth [3,4] and aquatic organisms [5].

One of the main sources of micropollutants in Europe is the discharge from municipal wastewater plants. About the same amount of the load comes from inputs through drainage from agricultural land treated with pesticides and about 20% from inputs from industrial activities. While in the European Union no regulation on upgrading of WWTPs to reduce micropollutant loads in surface water has been approved yet, Switzerland is the only country to have a national strategy against micropollutants in water, which will start on 1 January 2016. The technologies that have been selected are mainly based on activated carbon (AC) treatment technologies and/or ozonation. For example, to reduce the release of micropollutants into the aquatic environment, two large-scale pilot treatments were tested in parallel for over more than one year [6]. Feed water was the effluent of the municipal WWTP of Lausanne, Switzerland. The treatments were: (i) oxidation by ozone followed by sand filtration (SF) and (ii) powdered activated carbon (PAC) adsorption followed by either ultrafiltration or sand filtration. Micropollutants were removed on average at a rate of over 80% compared with raw wastewater, with an average ozone dose of 5.7 mg L^−1^ or a PAC dose between 10 and 20 mg L^−1^.

Depending on the chemical properties of the substances, either ozone or PAC performed better. Dissociated moieties have a higher electron density and thus are more reactive towards ozone [7]. PAC efficiency is improved for hydrophobic or positively charged compounds [6].

A treatment with granular activated carbon (GAC) filtration has been recently considered as a potential alternative and is already applied to a full-scale WWTP in Germany [8]. The efficiency of GAC filtration in removing micropollutants depends on the compound and the frequency of GAC regeneration/replacement [9].

Although ozonation is almost always the cheapest solution, for drinking water resources it is not the best choice [10]. Toxic oxidation byproducts can form during ozonation of wastewater, such as carcinogenic bromate, nitrosamines or formaldehyde [11]. PAC ultrafiltration treatment is considered to be the most suitable option, enabling good removal of most micropollutants and macropollutants without forming hazardous byproducts, the strongest decrease in toxicity and a total disinfection of the effluent [6]. However, active carbon must be removed from the purified water to minimize the losses of AC in the effluent. PAC is typically a single-use product, and at the end of life is burnt, while GAC is thermally regenerated and reused [12]. Energy consumption and the CO_2_ footprint are greater than ozone.

For large-scale municipal applications, current photocatalytic water treatment systems are less attractive because they are more time-consuming and have higher costs than other existing advanced oxidation techniques such as UV/H_2_O_2_, O_3_/H_2_O_2_ and UV/O_3_ technologies [13,14]. However, for water treatment in select niche applications, photocatalysis still retains substantive and unique benefits [15]. For example, photocatalysis enables not only oxidation but also reduction, presenting relatively untapped opportunities to reductively remove oxyanions, such as nitrate [16], chromate [17] and redox-active metal ions, such as Ag^+^ [18]. The ability to reduce oxygen to form H_2_O_2_ by select photocatalysts, such as g-C_3_N_4_, may also become a useful approach to produce AOP precursors on site [15,19].

**Figure 1:**
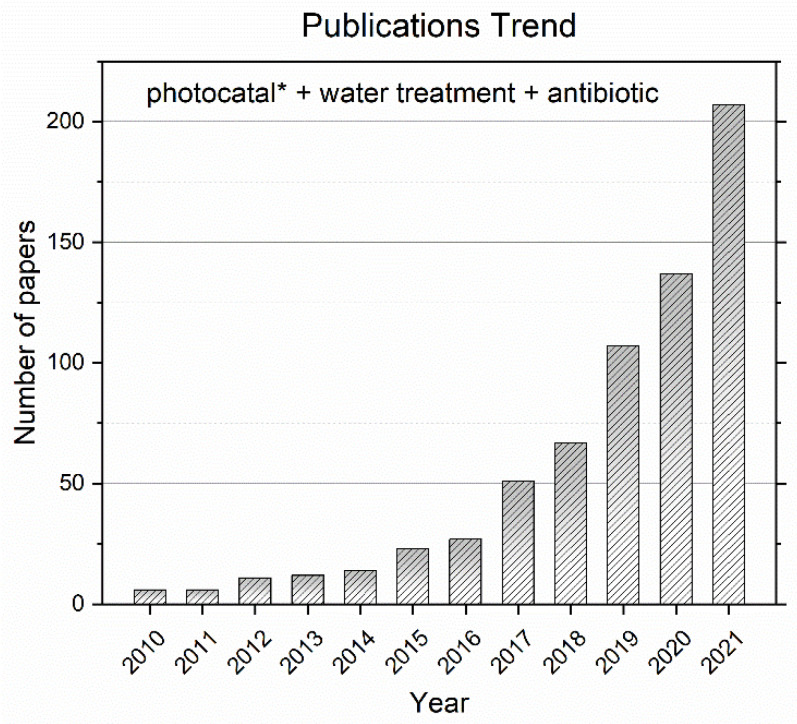
Publications trend: Web of Science results for the number of yearly publications on photocatalytic water treatment of antibiotics from 2010 to 2021.

Figure 1 shows the publication trend in the scientific literature found using “photocatal* + water treatment + antibiotic” searching terms. The trend shows an increasing interest in photocatalytic wastewater treatment, but generally the studies are not about real wastewater [20]. This feature limits further development of application of photocatalysis for real wastewater treatment. We therefore considered a survey of these publications to be of interest, focusing on one specific pollutant, namely oxytetracycline (OTC), a widespread antibiotic, commonly used for both humans and animals and therefore frequently used as model pollutant in the assessment of the effectiveness of treatments. OTC is a well-known persistent organic pollutant (POP), and a number of different treatments have been proposed for its removal from wastewater, ranging from classical methods, such as adsorption or chemical oxidation, to advanced decontamination methods, such as photocatalysis.

### 1.1. Method

Due to the huge number of recent papers on the subject, we decided to focus on photocatalytic methods of removal and to only take into consideration those papers meeting criteria that would ensure reproducibility of the results. We chose to take into consideration only those papers where all the following data are clearly stated: (i) OTC concentration; (ii) catalyst concentration (where applicable); (iii) required time; (iv) characteristics of the light source; and (v) percentage of removed pollutant.

### 1.2. Occurrence of Oxytetracycline in Wastewater

The worldwide market of antibiotics and antimicrobials was estimated at USD 4.7 billion in 2021, and the tetracycline segment accounts for the largest market share [21]; for instance, it was reported that approximately 248,000 tons of antibiotics are manufactured in China each year and that approximately 52% of them are used as veterinary antibiotics. Approximately 40–90% of the antibiotics used in humans and animals are excreted in feces or urine, either in their active form or as glycoconjugates, readily transformed back into the parent form [22,23]. In one study, 17 antibiotics were detected in 50 samples of livestock manure and compost in 8 provinces of China [24], and among them the concentration of oxytetracycline was the highest, reaching 416.8 μg kg^−1^. Consequently, due to leakage or to the use of animal manure as fertilizer, large amounts of residual antibiotics have been detected in soil and groundwater. In Europe, while human use of antibiotics is increasing, their use as growth promoters in animal husbandry is decreasing due to regulations, with particular regard to tetracyclines. Between 2010 and 2013, a 7.9% decline in antibiotics sales used in veterinary medicine was observed in 23 European countries. A north–south gradient of veterinary antibiotics use is observed, with the lowest consumption in northern Europe (85 mg/PCU on average in 16 countries, where PCU, Population-Corrected Units, is defined as number of livestock animals × estimated weight at treatment) and the highest consumption in southern Europe (260 mg/PCU in Spain, Italy and Cyprus). The group of tetracycline antibiotics accounts for approximately 37% of consumption in the 26 EU/EEA countries considered [25]. In any case, their occurrence in groundwater is not detected at alarming levels, as can be seen, e.g., in studies on wastewaters in Luxembourg [26] and Slovakia [27].

**Figure 2:**
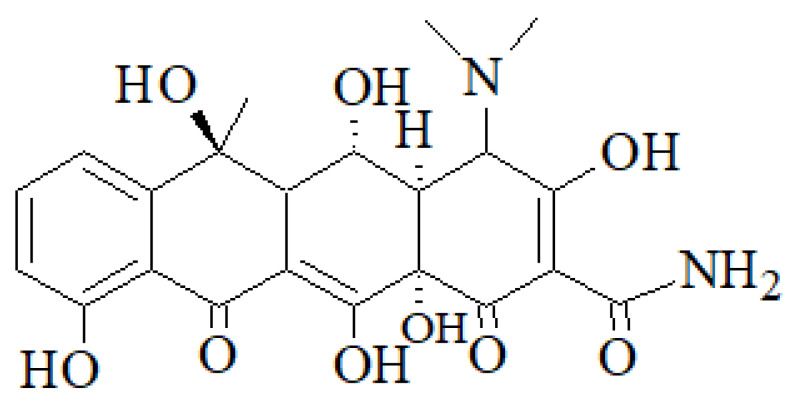
The structure formula of OTC.

We chose to focus our attention on OTC (Figure 2), a broad-spectrum antibiotic belonging to the class of tetracyclines, patented in 1949 [28], which has been widely used for the treatment of infections caused by strains of Gram-negative and/or Gram-positive bacteria in both humans and animals. Presently, it is mostly used for the treatment of acne and of infections caused by *Chlamydia* and *Mycoplasma* pathogens.

In addition, it is used in the treatment of livestock, both as a growth promoter factor and for the prevention of disease in cattle, poultry and fish. It is also used to control bacterial diseases of bees and tree fruits [28].

To date, oxytetracycline is widely found in natural water systems at about 10–100 ng L^−1^ levels [29,30,31].

The occurrence of OTC in water may derive from several sources:

**Effluents of sewage treatment plants:** Tetracyclines are among the most frequently detected antibiotics in wastewater. Here, OTC, which mostly comes from human consumption, appears to be mostly adsorbed in the sludge [32], since tetracyclines have complexing properties and can easily bind to calcium and similar ions, thus forming stable complexes, which can adhere to suspended matter or sewage sludge. In addition, standard degradation techniques sometimes fail to completely mineralize OTC in water treatment plants [33].

**Cattle manure dispersed in the fields:** Since only part of the administered drug is metabolized in the body, the rest being excreted in its active form [23]; a non-negligible amount can be found in manure. Sorption of OTC to manure is rather high, and it is believed to be influenced by ionic binding to divalent metal ions such as Mg^2+^ and Ca^2+^ as well as to other charged compounds in the matrix. However, the binding of oxytetracycline to soil is stronger than its binding to manure, most likely due to the strong metal complexes formed between soil, metal ions and OTC [32].

**Aquaculture farms:** Here, antibiotics are administered either in the feed or by addition to the water. Most of the non-metabolized drugs are adsorbed by the sediments, where a part is degraded, while the rest may be slowly released into the open water [34].

**Effluents of the pharmaceutical industry:** This source could release even very high values of pollutants: one instance was cited for an OTC production plant in China with an average concentration of 20 mg L^−1^ in the effluents [35]. As a result, the OTC concentration in the receiving river, as high as 641 ± 118 μg L^−1^ at the discharge point, decreased to 377 ± 142 μg L^−1^ at the last sampling site, a distance of approximately 20 km from the discharge point. However, this being a typical case of point source, dealing with it would be much easier than in the case of non-point discharges. Some of the remediation methods described below have been tested on high concentrations of OTC (e.g., [36,37]) and are therefore appropriate for dealing with this particular aspect.

Hence, there is growing interest in the fate of OTC in the environment and in methods for its removal from wastewater treatment systems. Besides standard methods of decontamination, advanced oxidation methods are increasingly being investigated.

## 2. Standard Processes of Removal/Degradation of OTC in Water

When released into the environment, antibiotics undergo a series of biological and non-biological degradation processes. However, it is difficult to achieve complete degradation of the antibiotics in the environment, and more often they will be degraded, over a rather long time, to form a series of metabolites which often show higher toxicities than the parent compounds. The degradation pathways of OTC include non-biological degradation, mainly consisting in photodegradation, oxidative degradation and hydrolysis and biodegradation, mainly by plants and microbes.

Photolysis is one of the most important degradation paths for OTC in the environment (see Section 2.4); however, it is relatively slow at low ambient temperatures. It can take place both by direct adsorption of light by OTC (mainly at wavelengths of 250–300 and 340–380 nm) or through the action of photosensitizers, such as NO_3_^–^, NO_2_^–^, CO_3_^2–^, Fe^2+^, Fe^3+^, NaCl and TiO_2_, which accelerate the process. The main factors affecting OTC photodegradation include pH, light conditions, moisture and metal ions, while biodegradation is mainly affected by temperature and pH [38].

**Figure 3:**
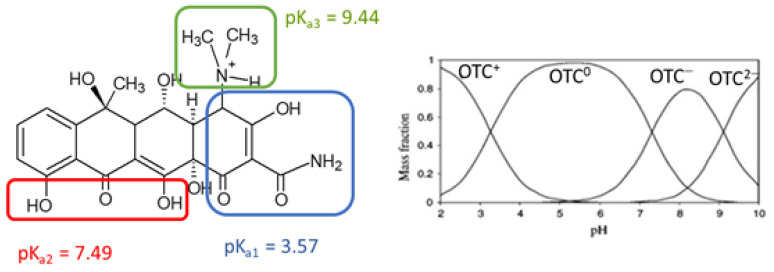
Ionization of OTC at various pH [39]. Reprinted with permission from: Kulshrestha, P.; Giese, R.F.; Aga, D.S. Investigating the Molecular Interactions of Oxytetracycline in Clay and Organic Matter: Insights on Factors Affecting Its Mobility in Soil. *Environ. Sci. Technol.* **2004**, *38*, 4097–4105. https://doi.org/10.1021/es034856q. Copyright 2004 American Chemical Society.

The importance of pH in all degradation pathways is related to the fact that the physico-chemical properties of OTC in water can vary with pH, since this molecule possesses three acidic groups and it can therefore be present in cationic, zwitterionic or anionic forms [39] (Figure 3).

OTC exists predominantly as a cation (OTC^+^) below pH 3.3, when the dimethylammonium group is protonated (pK_a1_ = 3.57) as a zwitterion, resulting from the loss of a proton from the phenolic diketone moiety (pK_a2_ = 7.49) between pH 3.3 and 7.3, as a single-charge anion (OTC^−^) from the loss of protons from the tricarbonyl system between pH 7.3 and 9.2 and as a doubly charged anion (OTC^2−^) from the loss of a proton from the phenolic diketone moiety, above pH 9.2 (pK_a3_ = 9.44) [39]. OTC is in any case very soluble in water between pH 1 and 10, but decontamination methods as well as analytical protocols must in all cases take into account the effect of pH, since, for instance, light adsorption and quantum yield in photochemical treatments depend on the mainly present ionization form [40].

OTC can be removed from water and sludge by a number of different decontamination techniques, among which adsorption on different materials, bioremediation by a wide variety of organisms or by enzymes and treatment with oxidizing agents such as ozone, hydrogen peroxide or persulfate, with or without catalysis, are the most studied and applied. While adsorption methods present the problem of disposal of the spent adsorbent, bioremediation and oxidation methods should take into account the possibility that the ultimate fate of the contaminant may not be complete mineralization but transformation in more or less noxious byproducts. In a very limited number of cases, the resulting products are identified and studied [41,42], while in other cases the overall toxicity is assessed by monitoring the effects on microorganisms [37].

### 2.1. Adsorption

Due to the complexing properties of OTC, this is a powerful medium for removal of this drug from water. The fact that OTC can be present in wastewater and soils as a cation, zwitterion or net negatively charged ion, depending on pH, complicates predicting its sorption characteristics and potential bioavailability and toxicity [43]. In addition, OTC adsorption in soils depends on the characteristics of the soil itself: soils with higher illite (a common non-expanding clay mineral) content and permanent cation exchange capacity have a higher OTC sorption capacity, but increase the availability of sorbed OTC, which can be released into the aqueous phase. Reversely, soil organic matter and soils characterized by the presence of clay, kaolinite or variable cation exchange capacity have a lower OTC sorption capacity, but decrease the release of sorbed OTC [43].

A wide variety of organic and inorganic materials, either natural, modified or synthetic, have been tested for OTC adsorption. Adsorption phenomena have been found to depend, besides pH, on temperature and on the presence of ions and other contaminants.

AC is one of the more widely used adsorption media, due to its capacity to adsorb relevant amounts of a wide variety of pollutants, and AC obtained by different sources and with different techniques has been used in several studies for the adsorption of OTC [36,44]. Though many waste materials from agriculture have been used as such as low-cost adsorbents, they have also been used as starting materials for the production of AC, whose effectiveness has been tested for the adsorption of OTC. For instance, AC was produced from cotton linter fibers [45] and from corn stalks [46]. In the first case, the adsorption capacity was 738.5 mg g^−1^; in the latter, the adsorption capacity, as well as the percentage of removal, were reported to vary widely based on temperature, pH, contact time and initial OTC concentration, with a maximum value of 522.6 mg g^−^^1^. A biochar was obtained from cauliflower leaves combined with natural attapulgite (a hydrated magnesium aluminum silicate with a layer chain structure) and FeCl_3_ [47].

Among natural materials, wastes from agriculture have been frequently exploited as low-cost adsorbents. Finely ground peanut shells were used as solid support for polyaniline, a conducting and electroactive polymer used to impart surface modifications to improve the natural material’s adsorption capacity [48]. Additionally, willow leaves, stems and roots have been used, as such and after desugarization, as an adsorbent medium, with promising results [49].

Some employed sorbent materials were based on carbohydrates; in one instance, OTC was removed from water by a hydrogel film composed of β-cyclodextrin–carboxymethylcellulose (β-CD/CMC) [50].

A wide variety of inorganic substances, such as clays, metal oxides and carbon, alone or in composite materials, were tested for the sorption of OTC. Among them, hydroxyapatite, both in the form of nanopowder [51] and in a mixture with silicates, proved effective at treating highly loaded water (25–100 mg L^−1^ OTC) [52].

Montmorillonite is a clay with a high surface area (700–800 m^2^ g^−1^) and a high cation exchange capacity (80–150 meq 100 g^−1^). Wet montmorillonite swells to five times its original volume in the dry state. Adsorption reactions can occur to a greater degree in montmorillonite than any other clay, so it has been used as model clay mineral for screening clay–OTC interactions [39]. It was also modified with iron (III) [53].

Zeolites were also employed, which work at an optimal pH between 7 and 8 and can be regenerated [54].

Graphene oxide (GO), a precursor for graphene preparation obtained through the strong oxidation of graphite, is extremely hydrophilic due to the presence of many polar functional groups on its surface; for this reason, it was used, among others, as an adsorbent in an aqueous medium. [55].

Composites of graphene, graphene oxide or reduced graphene oxides were also synthesized by various research groups and tested for OTC adsorption [56,57].

Magnetic microspheres with a magnetic core and a bimetal oxide shell (ZnO−Co_3_O_4_) were synthesized, which retained the functions of the magnetic nanoparticles but also provided activity conferred by the bimetal oxide shell. [58].

Tetracycline antibiotics (TCs), including OTC, adsorb strongly to aluminum oxide (Al_2_O_3_), and the surface interaction promotes structural transformation of TCs. [59].

### 2.2. Bioremediation

In the quest for greener methods of water decontamination, the bioremediation option has been widely explored, exploiting different kind of organisms. Selected bacterial strains, usually obtained through enrichment of activated sludge from wastewater treatment plants, were used in model systems [60,61]. Studies on biodegradation by bacteria conducted in model systems suffer the problem of the survival of microorganisms when introduced to soil or manure, since the number of introduced microorganisms would often decrease shortly after inoculation. In addition, the problem of OTC metabolites has to be addressed, some of which could be even more noxious than the parent antibiotic.

Microalgae have been used for bioremediation [62] with a removal capacity of up to 97%. Degradation of OTC and its metabolites has also been suggested to be carried out by earthworms [63], which also increase nitrification of the soil. In this latter case, two main degradation products, 4-epioxytetracycline and 2-acetyl-2-decarboxamido-oxytetracycline, were identified.

Enzymes, such as laccases or peroxidases, isolated from natural bacteria, were also used to treat contaminated water in vitro. The enzymes may be immobilized [64] or simply added to the water sample together with hydrogen peroxide and a hydrogen donor [65].

### 2.3. Oxidation

Advanced oxidation processes (AOPs) are environmentally friendly technologies for degrading recalcitrant pollutants. They can generate in situ highly active oxidants, such as hydroxyl radical O•H, which can unselectively degrade persistent organic pollutants. Several oxidizing agents were used for OTC degradation, with or without catalysis; among them, the most used are peroxides, such as H_2_O_2_ or persulfate and ozone.

Since ozone is a widely used chemical for disinfection, it was tested for OTC removal. Ozone was proposed firstly as a technique for the partial removal of OTC from highly loaded pharmaceutical wastewater [37]; according to these authors, ozonation should be used to convert OTC into more biodegradable intermediates for subsequent mineralization by cheaper biological processes. However, later the kinetics of the process was studied in detail [66], and it was found that, by using an aqueous ozone solution in the presence of *tert*-BuOH as a hydroxyl radical scavenger, 99.9% removal of OTC can be obtained at exposures well below those used for disinfection.

In one study, ozonation was also compared to Fenton oxidation by H_2_O_2_ in an OTC slurry obtained from extraction from manure. [67] Different concentrations of FeSO_4_ and H_2_O_2_ solutions were added to the acidified manure slurry; on the other hand, the manure slurry was ozonated in semi-batch mode with a continuous flow of the ozone and oxygen gas mixture. Both techniques provided more than 90% OTC removal.

An electro-Fenton process has also been proposed [68], which employs a NaOH-activated graphite felt electrode at pH 3.

Persulfate, S_2_O_8_^2−^, was used alone and with different catalysts. In one study, thermo-activated persulfate at a temperature range of 40–70 °C was found to degrade OTC rather quickly, though it is reported that OTC is thermally unstable, and approximately 40% of OTC was removed in 30 min, even in the absence of persulfate; however, further degradation only took place in the presence of persulfate. [69] Persulfate can also be activated by the combined action of heat and Fe^2+^ ions [70], with the effect of heat (up to 75 °C) being more important than that of Fe^2+^ ions. In another instance [71], the composite catalyst Co_3_O_4_/(carbon nanotubes, CNTs), due to the synergistic effects between highly active Co oxide and CNTs, was used for persulfate activation and OTC degradation, with outstanding catalytic performances in a wide range of pH from 3.0 to 9.0. The catalyst can be reused five times and can be separated conveniently by a magnet.

### 2.4. Photolysis

**Figure 4:**
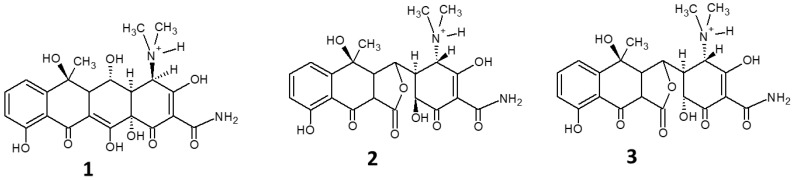
Structures of the main hydrolysis products of OTC, 4-epioxytetracycline (**1**), α-apooxytetracycline (**2**) and β-apooxytetracycline (**3**).

OTC undergoes direct photolysis as the main elimination pathway in surface waters. Photolysis increases with increasing temperature and with decreasing OTC concentration. The efficiency of removal depends on pH, since OTC can assume four differently ionized forms at different pHs, each with a different UV-vis absorption spectrum. [40] Photolysis was also studied in an aqueous abiotic environment in comparison with hydrolysis [72]. While the latter accounts for 20% OTC degradation, forming 4-epioxytetracycline (**1**), α-apooxytetracycline (**2**) and β-apooxytetracycline (**3**) (Figure 4) as the main hydrolysis products, photolysis is responsible for the remaining 80% of OTC degradation.

Photolysis seems to be the dominant degradation pathway of oxytetracycline in shallow, transparent water [73], and it is enhanced in the presence of Ca^2+^ ions; however, in real systems such as aquaculture plants, it is likely that OTC is mostly deposited in the sludge at the bottom, thus remaining unaffected by solar irradiation [72]. The direct photolysis parameters were studied in the open air under sunlight [73], in the absence of oxygen [40] and in the presence and absence of aquatic plants [74], in order to clarify the role of the different parameters influencing the process.

Direct photolysis with UV-C radiation was thoroughly examined in comparison with AOPs where irradiation is combined with the action of an oxidizing reactant such as H_2_O_2_ or persulfate, S_2_O_8_^2−^. This study [75] evidenced that OTC degradation, though slower than in AOPs, takes place within 30 min even with simple UV irradiation at 254 nm. However, this process leads to the formation of organic intermediates, which fail to mineralize completely; AOPs are more effective than direct photolysis for mineralization.

Oxygen nanobubbles (gas cavities in the aqueous solution with diameters of less than 1 μm) are being considered as a useful technology for water treatment and disinfection; in one study, they were added to a photoreaction system to improve the photodegradation efficiency of OTC under visible-light irradiation [76]. The efficiency of photodegradation depends on the size of the nanobubbles; hydroxyl radicals were identified as the dominant active species responsible for OTC degradation. In simply aerated solutions, the photodegradation efficiency was about 40% after 4 h of reaction, but in the presence of oxygen nanobubbles, the photodegradation efficiency increased to 60%.

### 2.5. Other Processes

A few other instances are reported in the literature for OTC removal from aqueous matrices, employing a variety of methods which do not fall within the above cited categories. Some of these are based on the use of various types of membranes. A membrane system including reverse osmosis and ultrafiltration was used for the pre-treatment of high load wastewater and was reported to reduce OTC concentration from more than 1000 mg L^−1^ to lower than 80 mg L^−1^ [77].

A piezocatalytic active membrane, based on a mixed matrix of exfoliated multi-flaw MoS_2_ nanosheets and polyvinylidene fluoride, was synthesized via an electrospinning technique [78]. This membrane was active in degrading OTC under ultrasonic irradiation in the dark. Mineralization of OTC was incomplete; a reaction mechanism and a series of intermediates were proposed. The membrane can be reused five times with only a slight decrease in efficiency.

Degradation of OTC was also obtained by a gas phase dielectric barrier discharge plasma reactor [79]. The generation of hydroxyl radicals, H_2_O_2_ and O_3_, in discharge plasma is responsible for the removal process, and strictly depends on the applied voltage. After 20 min of discharge treatment, approximately 93.4% of OTC was removed under the experimental conditions; however, only half of the initial OTC was mineralized or degraded to small molecules during this time. Some possible intermediates of the reaction are proposed.

## 3. Photocatalytic Degradation of Organic Pollutants

**Figure 5:**
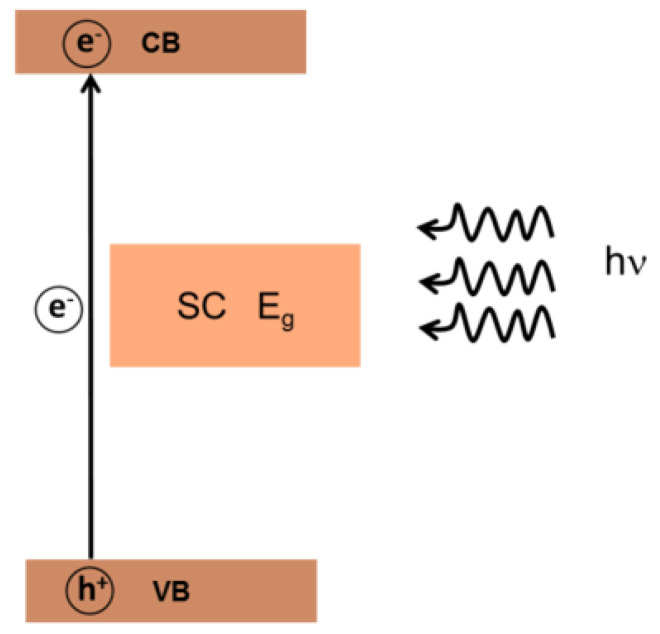
Schematic representation of the formation of excited electron−hole pair.

Photocatalytic oxidation is considered a promising alternative to the conventional methods of organic pollutant degradation. Using suitable catalysts, most organic pollutants can be completely mineralized to carbon dioxide under UV or visible-light irradiation. In the photocatalytic process, a chemical reaction is initiated when a semiconductor (SC) photocatalyst is irradiated by light with an energy that matches or exceeds the band gap energy of the semiconductor, resulting in excited electron–hole pairs [80]. Electrons are promoted from the valence band (VB) to the conduction band (CB), and holes remain in the VB (Figure 5).

From a thermodynamic point of view, an acceptor A can be photocatalytically reduced by CB electrons ($e_{\mathrm{CB}}^{-}$) if its redox potential is more positive than that of the $e_{\mathrm{CB}}^{-}$, and a donor D can be oxidized by VB holes ($h_{\mathrm{VB}}^{+}$) if its redox potential is less positive than that of the $h_{\mathrm{VB}}^{+}$. Electrons and holes migrate to the catalyst surface and initiate the redox reaction that can be applied to the degradation of an organic compound. The processes can be summarized by the following equations:

(1) $$\mathrm{SC}+\mathrm{hv}\;\to {\;e}_{\mathrm{CB}}^{-}+h_{\mathrm{VB}}^{+}$$

(2) $$e_{\mathrm{CB}}^{-}+A\;\to {\;A}^{•-}$$

(3) $$h_{\mathrm{VB}}^{+}+D\;\to {\;D}^{•+}$$

The photodegradation of pharmaceuticals under photocatalytic conditions is already carried out efficiently through the use of titanium dioxide (TiO_2_) and UV light. However, in wastewater treatment plants the application of AOP systems utilizing the TiO_2_ photocatalyst is infrequent, limited by a somewhat low photonic efficiency of the technology and by the use of energy-consuming ultraviolet (UV-A) lamps as a radiation source. To achieve a wider application of AOPs combined with photocatalysis, an efficient catalyst should be used, which could be activated with visible light. A synergistic effect is demonstrated when photocatalysis is coupled with other AOP technologies, such as ozonation, microwave or ultrasound treatments, although in some cases cost issues might arise [81].

### 3.1. Photocatalytic Oxidation of OTC Using Pure TiO_2_

TiO_2_ is a semiconductor photocatalyst. It is used for the removal of water pollutants due to its good chemical and environmental stability and the strong oxidizing power of the holes generated in the photocatalyst under UV irradiation [82,83]. Rutile, anatase and brookite are the three common crystalline polymorphs of TiO_2_. The anatase phase is the low-temperature stable form, and it exhibits higher photocatalytic behavior for oxidation processes as compared to the other polymorphs. TiO_2_ anatase, due to its wide band gap of E_g_ = 3.2 eV, can only absorb UV light, which is less than 5% of solar light.

The photocatalytic performance of pure TiO_2_ for OTC degradation under simulated solar irradiation is reported in few studies, as presented in Table 1. TiO_2_ P25 Degussa [84,85,86] significantly outperformed both other TiO_2_ powders [87] and TiO_2_ nanoflowers [88]. Colored TiO_2_, prepared by incorporating Ti^3+^ and oxygen vacancies in TiO_2_, is capable of harvesting visible light because of band gap narrowing. Recently, Singh et al. reported that mesoporous dark brown TiO_2_ shows good sunlight-induced photodegradation activity towards OTC-HCl molecules [89].

### 3.2. Photocatalytic Oxidation of OTC Using Heterogeneous TiO_2_-Based Photocatalysts

Practical applications of pure TiO_2_ are limited by the requirement of UV irradiation, which results in a low solar quantum efficiency and a high recombination rate of electron–hole pairs [90]. Several strategies adopted to implement the performances of TiO_2_ catalysts include doping TiO_2_ with metal and/or non-metal elements, surface functionalization, composites and nano-heterojunction.

Metal and/or non-metal doping has shown promising results in narrowing the band gap of doped TiO_2_, extending the light absorption properties of TiO_2_ into the visible-light region. Moreover, the electron–hole recombination is diminished. Few studies report OTC photodegradation by metal-doped TiO_2_. A nanocrystalline Co-B co-doped TiO_2_/SiO_2_ film removed 37% of OTC in 100 min under visible-light irradiation [91] (see Table 2).

The nanocrystalline Co-F co-doped TiO_2_/SiO_2_ film exhibited a band gap of 2.34 eV and removed 42% of OTC in 40 min starting from an unusually high OTC concentration (100 mg L^−1^) [98]. The N-TiO_2_/graphene film removed about 62% of OTC in 160 min [102].

Among surface nanostructures Ag, Au and Cu metal deposition on TiO_2_ is effective in suppressing the recombination of photogenerated electrons and holes in TiO_2_ and extending the activity into the visible-wavelength range [90,110]. As an example, Ag-decorated TiO_2_ samples demonstrated enhanced photocatalytic activity for the degradation of OTC under UV–visible-light illumination compared to that of pure TiO_2_. The sample containing 1.9 wt% Ag showed 100% removal of OTC under both UV (in 60 min) and visible-light (in 180 min) irradiation [92]. Au- and CuS-decorated TiO_2_ [94] removed 96% of OTC in 60 min under simulated solar irradiation.

Generally, it is possible to describe a composite as being constituted by a “matrix”, acting as a binder, and a “filler” which is added in the form of particles, fibers, etc. Nanocomposite materials exhibit different physical properties than any of the components separately, due to interfacial interactions of their phases and to the effect of the nanoparticles’ size on the concentration in the matrix. Nanocomposite catalysts, in which a phase consists of TiO_2_ anatase, have been extensively studied in recent decades with two main objectives: (i) to improve the photocatalytic reactivity and (ii) to obtain a TiO_2_-based photocatalyst which works under visible-light irradiation. A three-dimensional Ag_3_PO_4_/TiO_2_@MoS_2_ composite degrades 90% of OTC in 24 min via the following mechanism: under visible-light irradiation, the photogenerated electrons in the CB of Ag_3_PO_4_ are transferred to the surface of TiO_2_@MoS_2_ heterostructures, and subsequently transferred into a solution [93].

Heterojunctions between metal oxides and TiO_2_ offer low-cost materials for high performing catalysts. TiO_2_-based nanocomposites, such as CdS/TiO_2_ [97], Co_3_O_4_/TiO_2_ [99], Co_3_O_4_/TiO_2_ with GO as substrate [99], Fe_2_O_3_/TiO_2_ [101], TiO_2_/5A [106,107], ZnO/TiO_2_ [108] and ZnO/TiO_2_/Ag_2_Se [109] have been investigated.

Poorer performing photocatalysts are: BiOCl on TiO_2_ hollow tubes (51% of OTC removal, 150 min) [95], TiO_2_ cladded with graphene oxide (99% OTC removal, 240 min) [105], TiO_2_@V_2_O_5_ nanobelt covered with the conjugated polymer polypyrrole (85% OTC removal, 120 min) [103] and BiVO_4_/TiO_2_/RGO (99% OTC removal, 120 min) [96]. Porphyrins are common photosensitizers for TiO_2_. They lead to the generation of singlet oxygen due to their absorption in the range of 400–450 nm and very large molar extinction coefficient [111,112]. Porphyrin-functionalized TiO_2_ nanomaterials appear as a species-specific promising photocatalytic system for the removal of OTC in water [100].

### 3.3. Photocatalytic Oxidation of OTC Using Visible-Light Active Semiconductor Oxides

**Figure 6:**
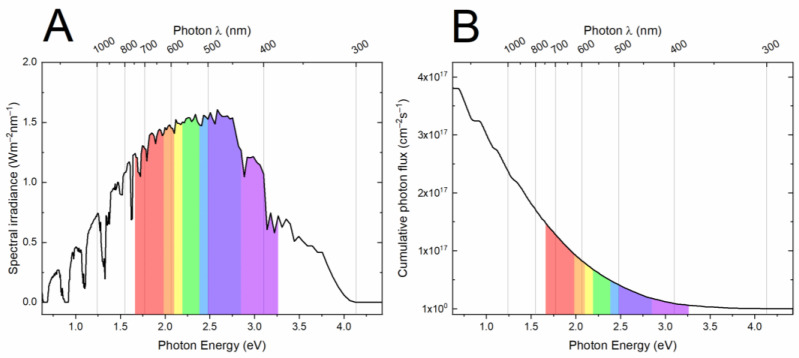
(**A**) Spectral irradiance. (**B**) Cumulative photon flux per photon energy (eV) and wavelength (λ). (Elaboration of data from https://www.nrel.gov/grid/solar-resource/spectra.html; source accessed on 4 April 2022).

The generation of an electron–hole pair in a metal oxide semiconductor using visible light is of major interest, given that UV light accounts for only a small part (about 5%) of the entire solar spectrum, while visible light has a much higher share (Figure 6). Metal oxides with band gap energies in the range of visible light energies were therefore pursued. These materials, in the form of nanostructured particles, nanosheets, nanorods and their combinations, are promising for wastewater treatment.

Metal oxides can be produced with large surface areas, different morphologies and with a wide range of band gaps, which are suited for photocatalysis. Among semiconductor oxides, various flavors of iron-based nanomaterials, such as magnetite (γ-Fe_3_O_4_) [113], hematite (Fe_2_O_3_) [101], LaFeO_3_ [114], zinc-oxide-based semiconductors [115], copper-oxide-based [116,117] nanoparticles and tungsten trioxide (WO_3_) [118], have been studied. Bismuth-based metal oxides, such as bismuth vanadate (BiVO_4_) [119,120], Bi_2_WO_6_ [112], Bi_2_MoO_6_ [121], BiYO_3_ [122] and combinations of the above, are gaining significant interest due to their narrow band gaps (2.4 eV), non-toxicity and photocatalytic activity under visible-light irradiation. A special class of materials is constituted by magnetic oxides, based on spinel structures, that allow for easy recovery of the catalyst after use and comprise NiFe_2_O_4_ [123,124] and MnFe_2_O_4_ [125].

A major issue with low-band-gap semiconductors is the fast recombination of the photogenerated electron–hole pairs, which easily annihilate due to the Coulombic force between electron and holes. When the recombination is fast, the direct oxidation/reduction or the generation of active species ($h^{+}$, O•H, $O_{2}^{•-}$, etc.) is limited. Moreover, in a single semiconductor photocatalyst, a small band gap, which assures a larger ability of harvesting light, limits the oxidation/reduction ability of the photocatalyst, which correlates with the energy difference between CB and VB.

Six different degradation pathways of OTC under visible-light active photocatalysts were proposed: demethylation, secondary alcohol oxidation, dehydration, hydroxylation, decarbonylation and deamination, as reported by Ye et al. [119].

#### 3.3.1. Simple Semiconductor Oxides

When single photocatalysts or basic composites are used, low degradation efficiency is usually reached, due to the fast recombination of electron and holes, such as in the case reported for Fe_2_O_3_ [101] and Fe_3_O_4_ and for Bi_2_WO_6_ [113], LaFeO_3_ [114] bare Bi_2_MoO_6_ [126] and MnFe_2_O_4_ [125], NiFe_2_O_4_ [123], or ZnO and ZnWO_4_ [115], and BiVO_4_ [119,127]. Mohan et al. [128] reported a 58% degradation for bare V_2_O_5_. In all the other reports, the degradation of OTC is below 50%. Nevertheless, excellent results are reported by Hernández-Arellano et al. [122] for a Ni-doped BiYO_3_ with a high surface area synthesized via a Pechini sol–gel method and calcined at 800 °C, which reached 97% OTC degradation in 300 min under Xe lamp illumination; a band gap as low as 2 eV has been reported for the pure cubic compound. Additionally, BiVO_4_ nanosheets with a thickness of 10 nm, synthesized by Xu et al., achieved a 95.8% degradation in 120 min [120]. He et al. [117] reported that CuCo_2_O_4_ nanoparticles removed 91.5% of the starting OTC in 180 min with the aid of some added H_2_O_2_ initiators. An outstanding 99% degradation was reported by Gautam et al. for a GSC (graphene–sand composite)/MnFe_2_O_4_ composite in 120 min under solar light [125]. It must be noted that the authors do not claim the formation of a heterojunction, but the oxide is supported on a high-surface-area material (GSC) obtained by pyrolization of sugar-coated sand that indeed doubles the degradation efficiency with respect to the bare oxide. Another hint that indeed some kind of electronic interaction must take place is the fact that the removal efficiency drops to 90% in the same conditions in bentonite (BT)/MnFe_2_O_4_ composites [125], where the BT support material is not expected to have any electronic activity. Promising results have been obtained by Li et al. [118] with a photochromic yellow-WO_3_ oxide, which exploited the photochromic interchange between the valence state +6 and +5 of tungsten to promote the formation of ROS species in the solution. The reported removal efficiency was 87.9% in 60 min. Beside pure oxides, S- and I-doped ones also have attracted interest. It is worth mentioning the work by Liu et al. [129], where iodine doping increased the removal ability of doped Bi_2_MoO_6_ microspheres up to 89.6% compared to 57.1% of the bare oxide under the same conditions (300 min, Xe 350 W lamp). It should be noted that the surface area also increases as a result of doping, which affects the adsorption capacity of the compound.

In all the other reported single semiconductor photocatalysts, the removal efficiencies lie in the range of 50 ÷ 80% degradation (see Table 3).

#### 3.3.2. Semiconductor-Oxide-Based Heterojunctions

To avoid the rapid recombination of charges that often occurs in low-band-gap oxide semiconductors, coupling two different semiconductor compounds with staggered conduction and valence bands is a viable solution. In this configuration, the photoinduced $e^{-}$ and $h^{+}$ end up accumulating on the two semiconductors and become able to promote oxidation and reduction of the target compounds. This can significantly enhance the energy conversion efficiency of the photocatalyst, sometimes at the expenses of the oxidizing ability with respect to specific species.

Heterojunctions where one of the photocatalysts is a semiconductor oxide have been largely studied. The counterparts are sometimes other oxides, such as in Raizada et al. [113] Fe_3_O_4_/Bi_2_WO_6_ or [115] ZnO/ZnWO_4_, or in Shi et al. [132], CuBi_2_O_4_/Bi_2_MoO_6_ or Liu et al. [133] β-Bi_2_O_3_@CoO, but more the use of a second (or a third) photocatalyst composed of graphene-like materials is implied, such as multi-walled carbon nanotubes (MWCN) [119], GO [134,135] graphitic carbon nitride (g-C_3_N_4_) [114,124], hexagonal boron nitride (hBN) [121] and polymeric carbon nitride (pCN) [130] (see Table 4).

Bismuth-based oxides are the most used in this context. For example, BiVO_4_ oxide, which by itself has a poor performance as a photocatalyst against OTC (degradation from 47.4% [119] to 61.1% [127] as reported in Table 3, depending on the reports), has been used to prepare heterojunction photocatalysts by coupling it with MWCN [119], Bi_2_S_3_ [136], Ag_2_S [140], Ag/AgCl [127], nitrogen-doped graphene quantum dots (N-GQD)/g-C_3_N_4_ [143] and AgI [144].

Ye et al. [119] prepared a BiVO_4_/MWCN catalyst that degraded nearly 90% of OTC within 60 min and withstood several 120 min cycles, degrading nearly 95% of OTC in each cycle. When used to make heterojunctions with Bi_2_S_3_, [136], the results are not as good: only 67% of OTC was removed from the solution after 16 h of illumination. As a counterpart, the same photocatalyst completely degraded Rhodamine B in 480 min (that is to illustrate that heterojunction photocatalysts sometimes fail to activate some specific catalytic reaction). More successful was the strategy of Dai et al. [127] in constructing a ternary heterojunction Ag/AgCl/BiVO_4_ photocatalyst prepared by in situ precipitation and photoreduction. They obtained a 97.6% degradation after 120 min of illumination with visible light (it must be pointed out that they used a 1000 W Xe lamp, far more powerful than in any other report in this review). The catalyst efficiency was ascribed to the presence of Ag nanoparticles on the surface, promoting a surface plasmon resonance effect. Interestingly, the photocatalyst showed only a slight decrease in efficiency in a wide pH range and was robust to the presence of nitrates and copper ions in the solution. Additionally, the mechanism was studied, and possible intermediate products were identified.

BiVO_4_ heterojunction with Ag_2_S was prepared by Wei et al. [140] following a somewhat similar strategy; Ag/Ag_2_S coupled with BiVO_4_ with 5% Ag enhanced both absorbance in the visible region of BiVO_4_, sped up the charge transfer and slowed down the recombination of electron–hole pairs. As a result, 99.8% of the initial OTC was removed in 150 min of visible-light irradiation. The investigation of the active species in the oxidation of OTH revealed that the main role was played by the generated $h^{+}$.

Guan et al. [144] decorated BiVO_4_ with silver iodide (AgI). The optimized catalyst with about 9% of AgI could remove 80% of the initial OTC, greatly enhancing the performance of bare BiVO_4_. The authors claim that a Z-scheme electron–hole transfer was at play, and it was responsible for the enhancement of the photocatalytic activity.

Last, a complex system Z-scheme heterojunction was proposed by Yan et al. [143], where BiVO_4_ was coupled with nitrogen-doped graphene quantum dots and g-C_3_N_4_. Despite the complexity, only 67% OTC was degraded after 120 min of visible-light exposure by a 250W Xe lamp. Nevertheless, ESR and trapping experiments elucidated the mechanism of the heterojunction, and the active species were identified in $O_{2}^{•-}$ and O•H radicals.

A Z-scheme heterojunction photocatalyst composed of β-Bi_2_O_3_@CoO was reported by Liu et al. [133], in which the bismuth oxide was prepared by a solvothermal method and CoO was grown in situ on the flower-like microstructured support. A degradation of 86% OTC after 120 min was obtained.

Other bismuth-based oxides have been reported. Bi_2_WO_6_ heterojunction with Fe_3_O_4_ was reported by Raizada et al. [113] to remove 71% OTC under 120 min of solar light illumination; this value increased up to 94% when the heterojunction was dispersed onto GSC. Yet, it is not clear if the role of GSC is only to increase the surface area, thus increasing absorption of OTC onto the catalyst, or if it has an active role in the generation or mobility of the photogenerated charges. The Bi_2_MoO_6_ heterojunction with hexagonal boron nitride (h-BN) was prepared by Du et al. [121]. In the optimized composition (50% h-BN), it degraded more than 95% of the starting OTC after 140 min of visible-light illumination. Even if h-BN has no catalytic activity in itself, its large surface area promoted the adsorption of OTC on the catalyst surface and the synergic interaction with the bismuth molybdate effectively separated the photoinduced charges. The authors also performed trapping experiments for elucidating the role of the diverse radical species and determined the prominent role of $h^{+}$ and $O_{2}^{•-}$.

A Bi_2_MoO_6_ n-type semiconductor coupled with a CuBi_2_O_4_ p-type one was prepared [132] with a peculiar structure made of nanosheets and nanorods, obtained via hydrothermal synthesis. Despite the perfectly staggered band structures and the refined microstructure, the degradation of OTC was limited to 74% in 60 min. However, more than 50% of it was completely mineralized.

Some interest has been raised by bismuth oxyhalides (BiOX, with X = F, Cl, Br, I). Thanks to their layered structure, the halide negative ions alternate with positive Bi_2_O_2_^2+^ layers, helping in the separation of the generated electrons and holes [145]. BiOX variously coupled with other oxides has been applied in the photodegradation of OTC under visible light.

Wen et al. [137] obtained the best performance by degrading 94% of the starting OTC in 90 min using a 30% SnO_2_/BiOI heterojunction photocatalyst, where SnO_2_ particles were grown in situ on BiOI nanosheets in different amounts. The importance of the in situ preparation and intimate contact between the two components is highlighted by the fact that a mechanical mixture of the same components using the same amount only degraded about 50% of OTC in the same experimental conditions. Additionally, using radical scavengers and inhibitors, the active species were determined to be $h^{+}$ and $O_{2}^{•-}$ radicals. Less advantageous was the coupling of BiOI with SrTiO_3_ by the same authors [139]; in this arrangement, about 85% of the OTC was removed from the solution in 90 min. Priya et al. [138] instead used BiOCl in a heterojunction with Bi_2_O_3_ and supported on large-surface-area materials, GSC and chitosan (CT). Good removal performances were obtained in both cases under solar light illumination (86 and 90% OTC degradation with GSC-supported and CT-supported photocatalysts, respectively). The authors underline the importance of OTC adsorption to the surface of the photocatalyst; indeed, bare Bi_2_O_3_/BiOCl, undispersed onto GSC and CT supports only degraded about 50% of the starting OTC in the same experimental conditions.

The investigation of heterojunction photocatalysts utilizing graphene-like materials is constantly increasing. In this last section, the OTC degradation papers are collected, where carbon nitride [114,124,130] or graphene oxide [134,135], were adopted in conjunction with semiconductor oxides.

g-C_3_N_4_ (see also Section 3.4.2 below), which by itself acts as a reduction photocatalyst, was coupled with LaFeO_3_ [114], in the fashion of the so-called Z-scheme heterojunction [146]. g-C_3_N_4_ remarkably acted both on the adsorption step and in the separation of photoinduced charges, increasing the removal efficiency of pristine LaFeO_3_ from 50 to 90% in 120 min of reaction under 40W LED light illumination. Sudhaik et al. [124] used g-C_3_N_4_ in a heterojunction with magnetic NiFe_2_O_4_ to obtain a remarkable 97% degradation in only 60 min under solar light illumination, and in 8 h, complete mineralization was also obtained. The authors also studied the effect of the initial OTC concentration and pH and concluded that the best conditions were those with high OTC concentrations (up to 24 mg L^−1^) and when OTC was in its zwitterionic form, at pH 5. Zheng et al. [130] also reported a 94% degradation using carbon nitride coupled with Ag_1.69_Sb_2.27_O_6.25_ in a 4:1 ratio in 120 min. Moreover, experiments with radical scavengers showed that the active species for the degradation were directly both the photogenerated e^−^ and $h^{+}$.

Graphene oxide was also investigated with CeO_2_/Fe_3_O_4_ [134] and with N-ZnO/CdS [135]. In the first case, the Fe^2+^/Fe^3+^ pair in Fe_2.8_Ce_0.2_O_4_ together with GO enhanced both adsorption of OTC and charge separation, yielding an 88% degradation of OTC in 120 min. N-doped ZnO and CdS were not as effective, degrading only 50% of OTC in 60 min, likely due to the lower efficiency of ZnO, given its larger band gap.

A somewhat different approach was attempted by Mohan et al. [141], who used a reduced graphene oxide composite with vanadium oxide (V_2_O_5_) coming from electronic waste recovery. The results for the 20%RGO/V_2_O_5_/Pt(1%) catalyst were an outstanding 99% degradation of OTC in 40 min, at the expenses of the experimental simplicity, by adding a O•H  initiator (H_2_O_2_) and 1% of a metallic Pt catalyst. Indeed, the same catalyst without Pt showed a diminished efficiency of 85% [128]. Yet, the authors interestingly applied the developed photocatalyst on real wastewater, reporting an 87% removal efficiency in real effluent conditions.

### 3.4. Photocatalytic Oxidation of OTC Using Graphene-Based Nanocomposites

#### 3.4.1. Graphene: Structure and Properties

**Figure 7:**
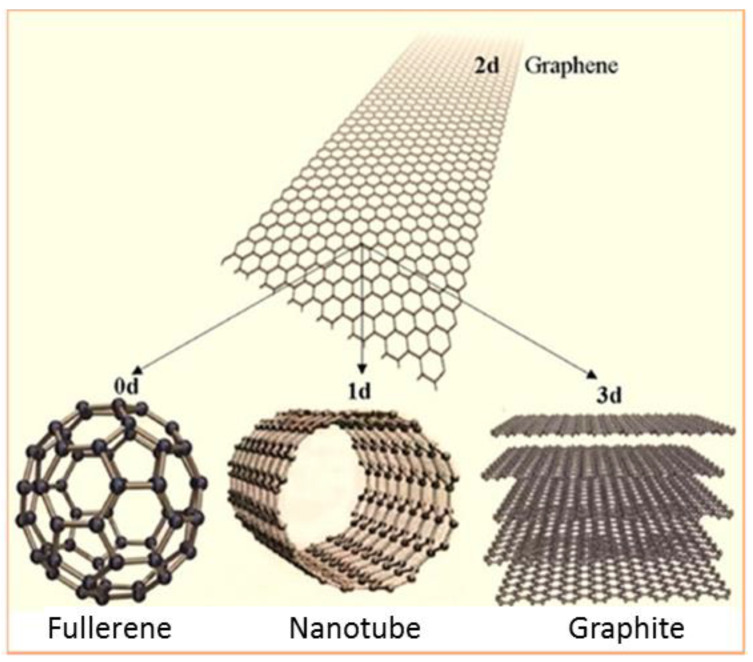
Different allotropes of carbon: graphite (3D); graphene (2D); nanotubes(1D); and fullerene (0D) Reprinted from reference [147].

Carbon has different allotropes, which can be categorized according to hybridization (sp, sp^2^, sp^3^), into zero-dimensional sp^2^ fullerenes, two-dimensional sp^2^ honeycomb lattice of graphene or three-dimensional sp^3^ crystals–diamond (Figure 7). Each allotrope shows distinct electronic and mechanical properties. Graphene consists of a single layer of carbon atoms that are bonded with a covalent sp^2^ bond with a single free electron, which accounts for the conductivity of graphene.

Graphene, fullerenes and CNTs, as novel nanomaterials, are attracting huge interest in different fields, such as physical, chemical and biomedical fields, thanks to the extraordinary physical properties, including extremely high thermal conductivity, excellent electrical conductivity, high surface-to-volume ratio, remarkable mechanical strength, biocompatibility and transparency to visible light as well as to UV and IR. Their considerable optical properties make them useful for photonic and optoelectronic applications [147].

Graphene is synthesized by various methods, such as mechanical exfoliation, liquid-phase exfoliation and chemical vapor deposition, which are implicated in restacking problems between graphene layers because of van der Waals forces. These issues have been resolved by surface modification with metal and metal oxide nanoparticles. Zinc (Zn), silver (Ag), gold (Au), platinum (Pt) and cadmium (Cd) are the most frequently used elements. Moreover, a large number of functional groups, such as carboxyl, hydroxyl ether and epoxide, are present on the surface of GO, which render it more dispersible in water and easy to use for composites design.

A N-ZnO/CdS composite was synthesized and subsequently incorporated on GO [135]. The obtained composite photocatalyst removes 50% of OTC in 60 min under visible-light irradiation. Recently, graphene quantum dots (GQDs) smaller than 10 nm have attracted attention because of their reservoir property, low toxicity and chemical inertness [148]. Moreover, it has been demonstrated that N- and S-co-doped GQDs (N, S-GQDs) show improved optical properties in comparison with undoped GQDs, which exhibit three excitation–wavelength–independent photoluminescence regions with emission peaks at 440 (blue), 550 (green) and 640 nm (red) at excitation wavelength intervals ranging from 340 to 420 nm, 460 to 540 nm and 560 to 620 nm, respectively. This means that each emission is related to a unified chromophore on the S,N-GQDs [149]. It was also shown that the association of N, S-GQDs and Au nanoparticles promotes near-infrared light (NIR) utilization. Based on these premises, ultrathin Bi_2_MoO_6_ (BMO) nanosheets were prepared and co-modified with N, S-GQDs and Au nanoparticles, whose catalytic activity was estimated through molecular oxygen activation for OTC degradation. This material shows 80% removal of OTC in 60 min [150]. Results are summarized in Table 5.

#### 3.4.2. Graphitic Carbon Nitride g-C_3_N_4_

**Figure 8:**
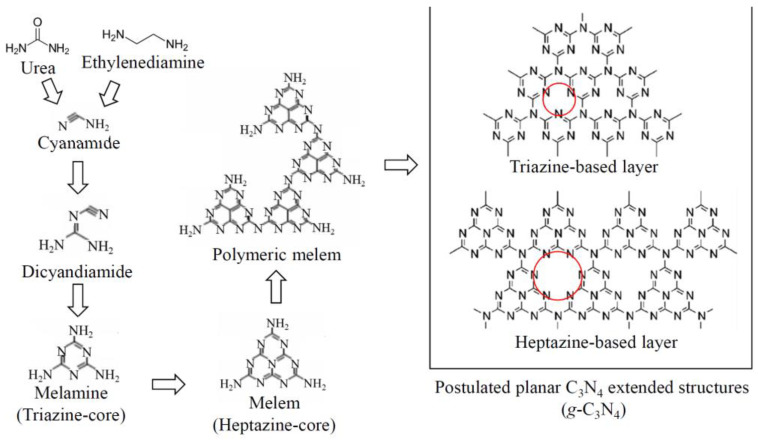
Structures of triazine-based and heptazine-based g-C_3_N_4._ Reprinted with permission from Ref. [164] Copyright 2019, Elsevier.

Recently, graphitic carbon nitride, a layered structure similar to graphene, has gained huge attention because of its unique physicochemical properties (e.g., material stability, thermal stability and narrow band gap) determined by S-triazine cores. The structure of g-C_3_N_4_ is often considered as an evolution of compounds with direct C-N bonding, such as urea and ethylenediamine, through cyanamide, melamine and their polymerized derivatives, resulting in either triazine-based or heptazine-based structures (Figure 8). The basic structural unit is either a triazine or heptazine core, both planar, so that the polymerized final product can be a layered structure. This is the reason why these polymerized products are called “graphitic” carbon nitrides.

The popularity of g-C_3_N_4_ can also be attributed to its environmental benignity, abundance, simple preparation process and low cost. Nevertheless, pristine carbon nitride (pCN) shows low electronic conductivity, limited visible-light absorption and relatively few surface-active sites. All these drawbacks have been resolved by different strategies, including heterojunction construction, heteroatom doping and nanostructural engineering. In particular metal oxides, metal sulfides, metal halides and carbonaceous nanomaterials (rGO, GO, carbon dots) are currently used as cocatalysts to improve the g-C_3_N_4_ photocatalytic performance [164]. Its photocatalytic activity under visible light attracts is of interest for studies on hydrogen evolution by water splitting and for pollutant degradation at room temperature.

The electron-rich “nitrogen pot” in pCN represents an ideal site for metal incorporation. Yang et al. [151], in 2020, developed a simple in situ growth strategy to anchor single-atom Co onto pCN by forming Co-O and Co-N covalent bonds. This implant not only extends optical absorption in the visible region but also facilitates electron transfer. These positive effects have improved the photocatalytic activity for OTC degradation. Different concentrations of Co, namely 0.29%, 1.28% and 2.52%, were tested, and the best result, albeit not satisfactory, was achieved with Co(1.28%)–pCN (18.3% OTC removal, 40 min). In 2020, Gou et al. [152] developed a strategy to prepare a novel oxygen-substituted ultrathin porous g-C_3_N_4_ nanosheet, and, as a result, this photocatalyst (OCN) showed enhanced ROS generation, leading to promoted OTC degradation (85.76% OTC degradation in 120 min).

Recently Ren et al. [153] and Viet et al. [154] conducted separate studies on OTC degradation using Ag-doped graphitic carbon nitrate as a photocatalyst, with 8% and 7% Ag by weight, respectively. The nanocomposite synthesized by Ren et al., Ag(8%)/g-C_3_N_4_ produced a degradation of oxytetracycline equal to 81% in 120 min. The photocatalyst investigated by Viet et al., Ag(7%)/g-C_3_N_4_, gave a better result, achieving 98.7% OTC removal in 120 min.

A 3 wt% NiSe/g-C_3_N_4_ photocatalyst, produced through an environmentally friendly hydrothermal method, showed remarkable photocatalytic activity, almost completely degrading OTC within 60 min (98.68% OTC removal) under visible-light irradiation [155]. These experimental results confirm that the use of highly dispersed NiSe nanodots enlarged the visible-light absorption range, potentiated charge carrier mobility and afforded rich active sites.

In 2019, Hong et al. [156] evaluated Br doping of g-C_3_N_4_ nanosheets, which showed photocatalytic activity equal to 75% OTC removal in 2 h.

Non-metal dopants are well-known to regulate the electronic structure and band gap of photocatalysts. Zhang et al. [157] improved the photocatalytic activity of g-C_3_N_4_ by co-doping of non-metal elements and morphologic regulation. They introduced B and P elements into the carbon nitride skeleton while decreasing the thickness via a thermal etching route. The obtained B,P-co-doped nanosheets (BPCNNS) had narrow band gap (2.61 eV) and thus utilized more visible light and ultrathin morphology to promote the separation and migration ability of photogenerated charges. The BPCNNS materials showed enhanced photocatalytic activity for removal of OTC in comparison with the un-doped, single-element-doped and co-doped bulk samples. A total of 71% OTC was removed after 120 min under visible light.

Among various types of semiconductors studied to augment the photocatalytic efficiency of g-C_3_N_4_ so far, polyaniline (PANI) was brought to the forefront due to its low cost, excellent environmental stability and high conductivity. The catalytic activities of the as-prepared catalysts with different amounts of PANI (3%, 5%, 7% and 10 wt%) were tested by using OTC as a model pollutant. In 100 min, 88% OTC removal was obtained [158]. Yang et al. [159], in 2020, synthesized a novel 2-hydroxy-4,6-dimethylpyrimidine (HDMP, 10 wt%) grafted polymeric carbon nitride photocatalyst (ACN) with a facile in situ keto–enol cyclization method. A total of 79.3% of the OTC was removed in 60 min.

The development of heterojunctions by coupling different semiconductors is also a popular strategy adopted by many researchers to improve photocatalytic activity. A novel B_4_NbO_8_Cl/g-C_3_N_4_ nanocomposite was synthesized using the hydrothermal method, and its photocatalytic efficiency was assessed under visible LED light irradiation, finding that it was much more enhanced than pristine B_4_NbO_8_Cl and g-C_3_N_4_; OTC removal was 87% in 60 min [160].

A facile ice-assisted ultrasonic method was developed to obtain a metal-free BPQD-loaded TCN (BPTCN) nanohybrid with a 1D tubular structure [161]. In this system, BPQDs were dispersed onto the tubular g-C_3_N_4_. A total of 81% of OTC was effectively removed in 60 min. A Au(6 wt%)/g-C_3_N_4_/CeO_2_ plasmonic heterojunction has been lately fabricated whose catalytic activity was carefully evaluated by the catalytic degradation of OTC (88% degradation in 150 min) [162].

Researchers have demonstrated that the construction of a heterojunction photocatalyst was more effective since the photogenerated electron–hole pairs were effectively transferred and separated. As a result, continuous efforts had been made to devise CN-based binary heterojunctions (CN/BiVO_4_, CN/BiOBr, CN/TiO_2_, etc.) However, the photocatalytic activity of these binary systems is still not sufficient for practical purposes because of the limited region of the visible-light response and the relatively lower photo-induced electron–hole pair separation efficiency. With the aim to further promote the charge separation and transfer characteristics, ternary system construction was developed. In 2017, a GO/Ag_2_CrO_4_/g-C_3_N_4_ (GO/ACR/CN) nanocomposite was fabricated through a facile precipitation route and employed for multiple pollutants’ degradation in experiments under visible-light irradiation [163]. Compared with ACR, CN, ACR/GO and ACR/CN, the GO/ACR/CN ternary photocatalyst showed enhanced photocatalytic performance. OTC removal reached 94.2% in 90 min.

## 4. Conclusions

In this review, the main aspects and recent advances in heterogeneous photocatalysis for the degradation of oxytetracycline antibiotics in water are presented. We chose to review only those papers where OTC concentration, catalyst concentration (where applicable), required time, characteristics of the light source and percentage of removed pollutant are clearly stated.

Photocatalytic oxidation of OTC using heterogeneous pure semiconductor oxides (TiO_2_ or metal oxides with band gap energies in the range of visible-light energies) is presented. To avoid the rapid recombination of electron–hole pairs that often occurs in low-band-gap oxide semiconductors, coupling two different semiconductor compounds with staggered conduction and valence bands is a good strategy. In this configuration, the photoinduced electrons and holes end up accumulating on the two semiconductors and become able to promote oxidation and reduction of the target compounds. Several solutions are adopted to enhance the performances of catalysts, including (i) doping the oxides with metal and/or non-metal elements, (ii) surface functionalization, (iii) composites and (iv) nano-heterojunction. Finally, a discussion on non-oxidic photocatalysts is also provided, exploring the application of graphene-based nanocomposites for the effective treatment of OTC.

TiO_2_ P25 Degussa [84] (95% OTC removal in 35 min) and Ag_3_PO_4_/TiO_2_@MoS_2_ [93] (90% OTC removal in 24 min) significantly outperformed other photocatalysts under UV irradiation. A g-C_3_N_4_/NiFe_2_O_4_ photocatalyst [124], showed remarkable photocatalytic activity in degrading OTC (97% OTC removal in 60 min) under sunlight irradiation. Furthermore, under visible-light irradiation, a 3 wt% NiSe/g-C_3_N_4_ photocatalyst [155] showed outstanding activity by almost completely degrading OTC within 60 min (98.7% OTC removal).

Existing decontamination techniques usually rely on adsorption of pollutants onto sorbent media, such as AC, which has to be eventually disposed of, or onto powerful oxidizing agents; however, these sometimes just partially oxidize persistent pollutants, yielding even more noxious byproducts. For large-scale wastewater treatment applications, current photocatalytic treatment systems are less attractive than other existing advanced oxidation techniques because they are more time-consuming and have higher costs. However, application of photocatalysis still retains significant and unique advantages since pollutants are often degraded to mineral end-products, and the degradation is achieved without transferring the pollutant (as is the case with conventional treatment technologies or AC) from one phase to another.

## Figures and Tables

**Table 1:** OTC photodegradation over pure TiO_2_ photocatalyst.

Photocatalyst	Light Source	(OTC) (mg L^−1^)	Catalyst (g L^−1^)	Removal (%)	Time (min)	Ref.
TiO_2_ P25 Degussa powder	Xe 1000 W	20	0.5	95	35	[84]
TiO_2_ P25 Degussa powder	Hg 24 W	5	1	100	180	[85]
TiO_2_ P25 Degussa powder	UVA 6 W	5	0.4	90	30	[86]
TiO_2_ powder	Solar UV	20	0.5	100	n.a.	[87]
TiO_2_ nanoflowers	Sunlight	0.5	1	80	60	[88]
Brown TiO_2_ spheres	Sunlight	5	5	≈50	80	[89]

**Table 2:** OTC photodegradation over heterogeneous TiO_2_-based photocatalysts.

Photocatalyst	Light Source	(OTC) (mg L^−1^)	Catalyst (g L^−1^)	Removal (%)	Time (min)	Ref.
Co-B co-doped TiO_2_/SiO_2_	Vis. light	5	film	37	100	[91]
Ag-decorated TiO_2_	30 W	0.5	0.5	100	60 (UV) 180 (Vis)	[92]
Ag_3_PO_4_/TiO_2_@MoS_2_	Xe 800 W	5	1	90	24	[93]
Au/CuS/TiO_2_	Xe 35 W	5	sheet	96	60	[94]
BiOCl/TiO_2_ hollow tubes	Xe 300 W	20	0.5	51	150	[95]
BiVO_4_/TiO_2_/RGO *	Xe 1000 W	10	n.a.	≈99	120	[96]
CdS/TiO_2_	Xe 300 W	40	1	81	50	[97]
Co^2+^/F^−^ co-doped TiO_2_-SiO_2_	W lamp	100	film	42	40	[98]
Co_3_O_4_/TiO_2_	Xe 300 W	10	0.25	76	90	[99]
Co_3_O_4_/TiO_2_/GO *	Xe 300 W	10	0.25	91	90	[99]
Cu-porphyrin-TiO_2_	UV 300 W	14	0.02	≈65	40	[100]
Fe_2_O_3_/TiO_2_	W 300 W	60	1	95	300	[101]
N-TiO_2_/graphene	Hg 250 W	30	film	≈62	160	[102]
Polypyrrole TiO_2_@V_2_O_5_	Xe 300 W	50	0.6	85	120	[103]
POPD/TiO_2_/fly ash	W 300 W	10	0.1	73	30	[104]
TiO_2_@GO *	Xe 500 W	20	0.6	99.4	240	[105]
TiO_2_/5A zeolite	UV 32 W	50	1	100	150	[106]
TiO_2_/5A zeolite	UV 32 W	50	0.5	100	210	[107]
ZnO/TiO_2_	Solar light	60	1	90.3	8	[108]
ZnO/TiO_2_/Ag_2_Se	Blue LED 36 W	5	film	≈55	360	[109]

* GO = graphene oxide; RGO = reduced graphene oxide. N.a.

**Table 3:** OTC photodegradation over semiconductor oxides.

Photocatalyst	Light Source	(OTC) (mg L^−1^)	Catalyst (g L^−1^)	Removal (%)	Time (min)	Ref.
Fe_2_O_3_/TiO_2_	W-Halide 300 W	60	1	≈50	300	[101]
Fe_3_O_4_	Sunlight ^1^	46	0.5	42	120	[113]
Bi_2_WO_6_	Sunlight ^1^	46	0.5	40	120	[113]
LaFeO_3_	LED 40 W	40	0.5	≈50	120	[114]
ZnO	Sunlight ^1^	46	0.5	41	120	[115]
ZnWO_4_	Sunlight ^1^	46	0.5	46	120	[115]
CuCo_2_O_4_	Xe 500 W	20	1.0	91.5	180	[117]
yellow-WO_3_	Au-Halide 500 W	20	1.0	87.9	60	[118]
BiVO_4_	Xe 500 W	10	0.25	47.4	60	[119]
BiVO_4_ nanosheets	Xe 500 W	20	1	95.8	120	[120]
Bi_2_MoO_6_	Xe 300 W	20	0.5	≈80	140	[121]
BiYO_3_	Xe 25 W	30	0.25	59	300	[122]
BiY_0.995_Ni_0.005_O_3_	Xe 25 W	30	0.25	97	300	[122]
NiFe_2_O_4_	Sunlight ^1^	46	0.50	<30	120	[123]
GSC/NiFe_2_O_4_ *	Sunlight ^1^	46	0.50	80	120	[123]
BT/NiFe_2_O_4_ *	Sunlight ^1^	46	0.50	69	120	[123]
NiFe_2_O_4_	Sunlight ^1^	46	0.50	65	60	[124]
MnFe_2_O_4_	Sunlight ^1^	46	0.50	<50	120	[125]
GSC/MnFe_2_O_4_ *	Sunlight ^1^	46	0.50	99	120	[125]
BT/MnFe_2_O_4_ *	Sunlight ^1^	46	0.50	90	120	[125]
Bi_2_MoO_6_ nanosheets	Xe 300 W	4.6	1	≈50	120	[126]
BiVO_4_	Xe 1000 W	20	1	61.1	120	[127]
V_2_O_5_	Xe 150 W	50	0.5	58	60	[128]
Bi_2_MoO_6_	Xe 350 W	10	0.6	57.1	300	[129]
I-Bi_2_MoO_6_	Xe 350 W	10	0.6	89.6	300	[129]
Ag_1.69_Sb_2.27_O_6.25_	Xe 300 W	16	0.5	63	120	[130]
S-CoFe_2_O_4_	W-Iodine 300 W	80	1	83	300	[131]

* GSC = graphene–sand composite; BT = bentonite. ^1^ light intensity 35 (±1)·10^3^ lx.

**Table 4:** OTC photodegradation over semiconductor-oxide-based heterojunctions.

Photocatalyst	Light Source	(OTC) (mg L^−1^)	Catalyst (g L^−1^)	Removal (%)	Time (min)	Ref.
Fe_3_O_4_/Bi_2_WO_6_	Sunlight ^1^	46	0.5	71	120	[113]
GSC/Fe_3_O_4_/Bi_2_WO_6_ *	Sunlight ^1^	46	0.5	94	120	[113]
g-C_3_N_4_/LaFeO_3_ (2%) *	LED 40 W	40	0.5	90	120	[114]
ZnO/ZnWO_4_	Sunlight ^1^	46	0.5	70	120	[115]
AC/ZnO/ZnWO_4_ *	Sunlight ^1^	46	0.5	96	120	[115]
Cu_2_O/α-Fe_2_O_3_ ^2^	Xe 300 W	10	n.a.^2^	73.3	60	[116]
MWCN/BiVO_4_ *	Xe 500 W	10	0.25	88.7	60	[119]
hBN/Bi_2_MoO_6_ *	Xe 300 W	20	0.5	95.3	140	[121]
g-C_3_N_4_/NiFe_2_O_4_ *	Sunlight ^1^	46	10	97	60	[124]
Ag/AgCl/BiVO_4_	Xe 1000 W	20	1	97.6	120	[127]
AgCl/BiVO_4_	Xe 1000 W	20	1	76.5	120	[127]
20%RGO/V_2_O_5_ *	Xe 150 W	50	0.5	85	60	[128]
pCN/Ag_1.69_Sb_2.27_O_6.25_ *	Xe 300 W	16	0.5	94	120	[130]
CuBi_2_O_4_/Bi_2_MoO_6_	Xe 300 W	20	0.30	74	60	[132]
β-Bi_2_O_3_/CoO	Xe 300 W	10	0.40	86	120	[133]
GO/CeO_2_/Fe_3_O_4_ *	Xe 220 W	30	0.8	60	120	[134]
GO/Fe_3-x_Ce_x_O_4_ *	Xe 220 W	30	0.8	88	120	[134]
N-ZnO/CdS/GO *	Xe 300 W	20	0.5	50	60	[135]
Bi_2_S_3_/BiVO_4_	Xe 500 W	200	1	67	960	[136]
30% SnO_2_/BiOI	Xe 300 W	10	1	94	90	[137]
GSC/Bi_2_O_3_/BiOCl *	Sunlight ^1^	46	0.5	86	120	[138]
CT/Bi_2_O_3_/BiOCl *	Sunlight ^1^	46	0.5	90	120	[138]
22% SrTiO_3_/BiOI	Xe 300 W	20	1	85.3	90	[139]
Ag/Ag_2_S/BiVO_4_	Xe 500 W	20	0.4	99.8	150	[140]
20%RGO/V_2_O_5_/Pt(1%) *	Xe 150 W	50	0.5	99	40	[141]
Ag/AgCl/CdSnO_3_	Xe 300 W	10	1	90	60	[142]

* MWCN = multi-walled carbon nanotubes; GSC = graphene–sand composite; BT = bentonite; AC = activated carbon; GO = graphene oxide; g-C_3_N_4_ = graphitic carbon nitride; hBN = hexagonal boron nitride; pCN = polymeric carbon nitride; CT = chitosan. ^1^ light intensity 35 (±1)·10^3^ lx. ^2^ electrode for electro-photocatalysis: 0.5 V bias applied.

**Table 5:** OTC degradation over heterogeneous g-C_3_N_4_-based photocatalysts.

Photocatalyst	Light source	(OTC) (mg L^−1^)	Catalyst (g L^−1^)	Removal (%)	Time (min)	Ref.
N-ZnO/CdS/GO *	Xe 300 W	20	0.5	50	60	[135]
N,S-GQDs/BMO *	Xe 300 W	10	0.1	81	60	[150]
Co(1.28%)–pCN *	Xe 300 W	20	0.3	18.3	40	[151]
OCN *	Xe 300 W	20	1	85.7	120	[152]
Ag(8%)/g-C_3_N_4_ *	Xe 300 W	20	1	81	120	[153]
Ag(7%)/g-C_3_N_4_ *	Xe 300 W	30	0.2	98.7	120	[154]
NiSe(3%)/g-C_3_N_4_ *	Xe 300 W	20	1	98.7	60	[155]
Br(15%)/g-C_3_N_4_ *	LED 38.5 W	10	1	75	120	[156]
BPCNNS *	Xe 300 W	15	1	71	120	[157]
PANI(5%)/g-C_3_N_4_ *	Xe 350 W	5	0.5	88	100	[158]
ACN *	Xe 300 W	20	0.3	79.3	60	[159]
B_4_NbO_8_Cl/g-C_3_N_4_	LED 18 W	20	1	87	60	[160]
BPQDs/g-C_3_N_4_ *	Xe 300 W	10	0.6	81	60	[161]
Au(6 wt%)/g-C_3_N_4_/CeO_2_	Xe 500 W	15	0.4	88	150	[162]
GO/Ag_2_CrO_4_/g-C_3_N_4_ *	Xe 300 W	10	0.2	94.2	90	[163]

* GO = graphene oxide; GQDs = graphene quantum dots; BMO = Bi_2_MoO_6_; CN, pCN = carbon nitride; OCN = oxygen-substituted ultrathin porous g-C_3_N_4_; g-C_3_N_4_ = graphitic carbon nitride; PANI = polyaniline; ACN = 2-hydroxy-4,6-dimethylpyrimidine grafted polymeric carbon nitride.
